# Genetic Factors Influencing Sperm Competition

**DOI:** 10.3389/fgene.2019.00820

**Published:** 2019-09-13

**Authors:** Alberto Civetta, José M. Ranz

**Affiliations:** ^1^Department of Biology, University of Winnipeg, Winnipeg, MB, Canada; ^2^Department of Ecology and Evolutionary Biology, University of California, Irvine, CA, United States

**Keywords:** sexual selection, sperm competition, male-male competition, genetic architecture, gene function, speciation

## Abstract

Females of many different species often mate with multiple males, creating opportunities for competition among their sperm. Although originally unappreciated, sperm competition is now considered a central form of post-copulatory male–male competition that biases fertilization. Assays of differences in sperm competitive ability between males, and interactions between females and males, have made it possible to infer some of the main mechanisms of sperm competition. Nevertheless, classical genetic approaches have encountered difficulties in identifying loci influencing sperm competitiveness while functional and comparative genomic methodologies, as well as genetic variant association studies, have uncovered some interesting candidate genes. We highlight how the systematic implementation of approaches that incorporate gene perturbation assays in experimental competitive settings, together with the monitoring of progeny output or sperm features and behavior, has allowed the identification of genes unambiguously linked to sperm competitiveness. The emerging portrait from 45 genes (33 from fruit flies, 8 from rodents, 2 from nematodes, and 2 from ants) is their remarkable breadth of biological roles exerted through males and females, the non-preponderance of sperm genes, and their overall pleiotropic nature.

## Introduction

Sexual selection refers to the differential ability among the members of one sex to compete for access to mates or to choose mates (Darwin, 1871). However, mating success does not guarantee successful reproductive output. Early work in insects (Parker, 1970) showed that females of a range of species mate promiscuously, storing the sperm from multiple males in their reproductive tract, which creates a postcopulatory competitive arena to fertilize the ova. Specifically, Geoff Parker’s work emphasized the value of any trait that increases the chances of a male’s sperm outcompeting those of others for fertilization, thus biasing reproductive output (Parker, 1970). Parker’s seminal work led to a myriad of related studies that confirmed the relevance of this mechanism in many other species that fertilize internally or externally (Smith, 1984; Birkhead and Møller, 1998).

Sperm competition is now recognized as a crucial mechanism of postcopulatory sexual selection that fuels trait evolution (Smith, 1984; Simmons, 2001), including the genitalia (Simmons, 2001), sperm attributes (Lupold et al., 2016), and behavior (Bretman et al., 2011). Additionally, sperm competition has the potential to reshape genome organization and functionality (Mueller et al., 2005; Hollis et al., 2014). For example, insect genes encoding male accessory gland proteins—ACPs, which are present in the male ejaculate and trigger a variety of postmating physiological responses in the female (Chapman et al., 1995; Tripet et al., 2003; Ram and Wolfner, 2007a)—exhibit some of the highest rates of duplication and loss (Mueller et al., 2005).

While the phenomenological aspects of sperm competition have been extensively examined (Birkhead and Møller, 1998; Simmons, 2001; Pizzari and Parker, 2009; Lupold and Pitnick, 2018), we still lack a precise knowledge about the underlying genetics that influence sperm competitive ability. This limited characterization prevents knowing what molecular processes are associated with sperm competition and what particular gene characteristics, such as their spatial expression patterns or the biochemical attributes of their encoded products, are the most relevant. Equally important, knowing the identity of the genes involved will result in a better understanding of how sperm competition might contribute to the speciation process, a link that has started to be explored, and, more in general, to gain valuable insights in the evolutionary dynamics of differential sperm competitive ability. For example, knowing the identity of the genes involved will enable molecular diversity studies both at the intra- and inter-specific levels, thus allowing evaluation of the relative role of different types of selection as well as genetic drift in driving the molecular evolution of sperm competition. Moreover, as some genes underlying sperm competition might act pleiotropically on a variety of traits, we will better understand correlated evolutionary responses that curtail or accelerate the rate of organismal adaptation.

Here, we focus on the genetic factors that influence the outcome of sperm competition in internal fertilizers. We pay special attention to genes for which there is a proven causal link to sperm competition phenotypes in experimental competitive settings in which the post-mating male–male rivalry is assessed. These phenotypes are either male or female traits whose variation results in biased reproductive output and include from differential paternity contribution, to altered sperm dynamics in the female reproductive tract, to sperm properties such as fertilization capability and viability. Importantly, these competitive settings are completely necessary in order to overcome assumptions about implications in sperm competition based on circumstantial evidence through gene attributes such as detected expression in reproductive tissues. In addition, these genes have been examined functionally, finding reasonable evidence of a direct relationship with sperm competition phenotypes. We also argue that, although the polygenic nature underlying sperm competitive ability and vast non-additive genetic factors have complicated the identification of relevant genes, the necessary experimental methodologies have become increasingly available. This is the case beyond the fruit fly *Drosophila melanogaster*—the species more extensively investigated until today—and rodents, as it will be discussed in relation to nematodes and ants. These inroads made in additional species stress that we are finally well poised to perform a systematic identification of genes that influence the outcome of sperm competition, as well as to dissect how these genes exert their effects, across a variety of phyla.

## Differential Sperm Competitive Ability

Polyandry is ubiquitous among internal fertilizers. For example, in natural populations of the white-footed mouse *Peromyscus leucopus*, it has been estimated that an average of 68% of the females are involved in multiple matings (Xia and Millar, 1991), and in different *Drosophila* species, the average number of different males fertilizing the offspring of a wild-caught female ranges from 1.4 in *Drosophila simulans* (Schlotterer et al., 2005) to 3.1 in *Drosophila mojavensis* (Good et al., 2006). Polyandry enables sperm competition, and there is evidence from different species of insects and mammals of wide variation in patterns of sperm utilization under competitive conditions (Møller and Birkhead, 1989; Simmons, 2001).

**Figure 1:**
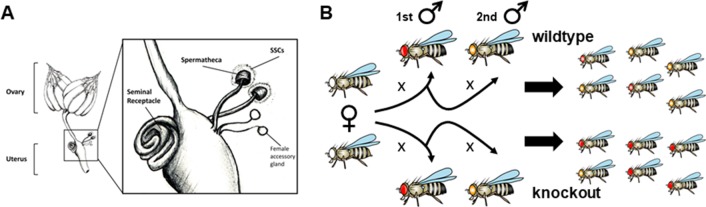
Assessing sperm competitive ability in *D. melanogaster*. **(A)** During copulation, sperm, as part of the ejaculate, are transferred to the uterus or bursa. Only one third of the ∼1,500 sperm transferred travel to the storage organs—a pair of spermatheca and a seminal receptacle (Miller and Pitnick, 2002; Manier et al., 2010). Sperm in storage can remain functional for long time periods, thus increasing the probability of sperm competition (Parker, 1970). The paired spermathecae are mushroom-shaped organs surrounded by spermathecal secretory cells (SSCs) while the seminal receptacle is a long tubular organ. Seminal receptacle and spermatheca function independently but there is communication between them (Schnakenberg et al., 2011). The two storage organs are differentially related to the sperm dynamics. The seminal receptacle is the first to get filled up by sperm, stores the highest sperm numbers, and is also the organ to first deplete sperm (Nonidez, 1920). The seminal receptacle is the most relevant organ in determining the fertilization set (Manier et al., 2010), *i.e.*, the set of sperm from different males that actually engages in sperm competition to fertilize the eggs (Manier et al., 2010). Fertilization occurs when the sperm released from the storage organs penetrate the anterior pole of the eggs through a particular structure named the micropyle, which happens upon the eggs have been pushed through the oviduct into the uterus (Nonidez, 1920). Sperm recruitment by the storage organs is influenced by the proper functioning of the female central nervous system (Arthur et al., 1998). Additionally, sperm recruitment rate by storage organs, modification, stability, and usage in the female reproductive tract are influenced by a diverse compendium of molecules present in the ejaculate and others secreted by the female spermatheca and accessory glands, the latter also known as parovaria (Schnakenberg et al., 2011; Sun and Spradling, 2013; Sirot et al., 2014). Displaced resident sperm and the excess of subsequent male sperm are ejected along with the mating plug—a seminal component-coagulated structure that forms in the female reproductive tract upon mating (Manier et al., 2010). Drawing by J.L. Sitnik from (Wolfner, 2011). **(B)** Typical double-mating assay to test for significant variation in sperm competitive ability between different experimental males. Females of known genotype mate consecutively with two males (1st, reference male; 2nd, experimental male), which carry alleles associated with particular markers (eye color in this case). Paternity identity for each offspring can be tracked based on the markers used and the relative contribution of each father to the total progeny number summarized through a score –P–. In this way, different experimental males can be compared against a common reference male (*e.g.*, knockouts against wildtype) to test if their corresponding P scores are statistically significantly different. The design shown, called offense, evaluates the ability of the sperm from the second male to outcompete first-male sperm from the female sperm storage organs, increasing the probability to fertilize the ova; P is denoted as P2. In a different version of this experimental design called defense, the experimental male is the first to mate while the reference male is second. In this case, the sperm ability to avoid being outcompeted by the sperm from the second male is evaluated and P denoted as P1. Alternatively, female contribution to sperm competition outcomes can be assessed by keeping both first and second male’s genotypes constant and varying the genotype of the females being tested. Further, this experimental design can be adapted to analyze sperm behavior in the female reproductive tract instead of differential paternity contribution. For this, both the experimental and reference males, or at least one of them, must carry transgenes for sperm monitoring purposes as they glow under the fluorescent microscope.

In many internal fertilizers, insemination and fertilization are temporally unlinked. Yet, females can store the sperm in specialized organs, increasing sperm lifespan and the probability of fertilizing the ova (Gist and Congdon, 1998; Shugart, 1988; Baer et al., 2006). There exist interspecific differences in the number of storage organs, their morphology, and storage capacity, resulting in differences in sperm dynamics, sperm usage, and ultimately in opportunities for sperm competition (Simmons and Siva-Jothy, 1998). For example, a study across 113 *Drosophila* species found that some of them use two different organs, the so-called spermatheca—in a variable number—and the seminal receptacle (**Figure 1A**), to store the sperm while other species use only one of the two (Pitnick et al., 1999). But even in many mammalian species, which typically lack female sperm storage organs and whose sperm are thought as short-lived, sperm competition has been widely documented (Møller and Birkhead, 1989).

Many details about sperm dynamics in the female reproductive tract in the context of sperm competition have been obtained primarily in insects (Simmons, 2001). In multiply-mated females of *D. melanogaster*, the most recent mate’s sperm displaces some of the previous males’ sperm from the female’s storage organs back into the bursa. Then, the displaced sperm and the excess of the most recent male’s sperm are ejected while the remaining sperm—the fertilization set—engages in competition (Manier et al., 2010). The outcome of sperm competition is heavily influenced by a wide range of sperm traits and environmental factors, which have been comprehensively reviewed elsewhere (Simmons and Fitzpatrick, 2012; Reinhardt et al., 2015).

## Detecting Sperm Competitive Ability and Mechanisms Inferred

Assaying differences in sperm competitive ability has relied largely on mating individual females with multiple males of differing genotypes, and then genotyping the progeny using molecular or morphological markers to determine what fraction is sired by any of the competing (also referred to as experimental) males (Boorman and Parker, 1976; Clark et al., 1995; Firman and Simmons, 2008; Hansen et al., 2015). These sequential mating trials can be performed in different ways. For example, the experimental males can be given access to the females in differing consecutive orders (House et al., 2007; Sutton et al., 2008). In another type of studies, the paternity contribution of the experimental males is inferred relative to a constant reference male. Thus, the relative contribution to the progeny of the experimental males (**Figure 1B**) is calculated when they are first (P1; defense) or second (P2; offense) to mate, respectively. These metrics capture different properties of the sperm when in competition, *i.e.*, to withstand or to promote displacement, respectively (Boorman and Parker, 1976; Clark et al., 1995), although they can also reflect factors unrelated to sperm competition. For example, last sperm precedence, when the last male fertilizes the majority of eggs (Boorman and Parker, 1976; Simmons and Siva-Jothy, 1998), can be caused by first-male sperm dumping prior to second mating or inviability of the eggs fertilized by the first male, resulting in a high P2 without sperm competition. These complications can be circumvented by incorporating corrections for viability, assays of copulation duration, and fecundity in non-competitive settings and by tracking females’ post-mating responses such as fidelity and egg-laying rates (Gilchrist and Partridge, 1997; Civetta et al., 2008; Levesque et al., 2010; Avila et al., 2011).

Ultimately, a better understanding of how sperm competition might occur can be gained by tracking patterns of sperm transfer, movement, and storage (Simmons and Siva-Jothy, 1998). The recent development of transgenics has resulted in this crucial increased level of resolution by allowing visual monitoring of sperm within the female reproductive tract. In *D. melanogaster*, the direct observation of the fate of fluorescently labeled sperm from different males within the reproductive tract of dissected females has provided unequivocal evidence for late-male sperm displacement (Civetta, 1999), a typical sperm competition outcome in many species (Boorman and Parker, 1976). In addition, sperm tracking provided no support for previous ideas of sperm incapacitation by showing that sperm functional parameters (motility and velocity) do not change due to long storage or upon female remating (Manier et al., 2010). In contrast, support was found for the role of sperm morphology and swimming as well as sperm storage and retention on sperm competition (Manier et al., 2010; Lupold et al., 2012; Lupold et al., 2013). Transgenic lines offer a unique opportunity to identify the interplay between genes—for example, once their functionality is perturbed—and sperm competitive phenotypes such as P1 and P2. We expect rapid translation of this approach to other species, as it has become evident by the development of sperm transgenics in the red flour beetle *Tribolium castaneum, in* the roundworm *Caenorhabditis elegans*, and in the flatworm *Macrostomum lignano* (Marie-Orleach et al., 2014; Hansen et al., 2015; Droge-Young et al., 2016).

Differences in specific sperm competition phenotypes can also be monitored *in vitro*. For example, in rodents, assays that examine the overall sperm fertilizing capability are commonplace. Thus, already capacitated sperm from different experimental males are co-incubated with free ova and subsequently the percentage of fertilized eggs by each type of male determined (Da Ros et al., 2008; Sutton et al., 2008). In other cases, the focus has been differential sperm viability. In insects such as the leafcutter ant *Atta colombica*, sperm viability is compromised by seminal fluid of rival males, an effect counteracted by the queen’s spermatheca secretions. *In vitro* assays using mass spectrometry analyses in which seminal fluids from focal and rival males are exposed to spermathecal fluids enable to quantify how sperm survival is affected (Dosselli et al., 2019). Overall, *in vitro* assays have increasingly become an important experimental strategy to inform about particular sperm competition phenotypes.

## Characterizing the Genetic Basis of Sperm Competition

The genetic architecture (number and identity of the genes, their genomic distribution, the quantification of their allelic effects, and their pleiotropic nature) underlying sperm competition remains largely elusive. This has been the consequence of at least three challenges. The first is the polygenic nature underlying sperm competition phenotypes as well as the difficulty to detect their underlying heritable genetic variance. This has prevented to identify *bona fide* candidate genes through approaches such as quantitative trait locus (QTL) mapping, arguably discouraging a more frequent use of this strategy. Also contributing to this scarcity of candidate genes, it is the fact that high-throughput surveys of mRNA and protein expression, as well as genomic data, have only recently become increasingly available across taxa. The second challenge has been the frequently no implementation of detailed phenotypic tests in competitive settings and the lack of functional analyses that could provide solid proof of the implication of a candidate gene in sperm competition phenotypes. The third challenge has been that, while sperm competition phenotypes P1 and P2 are relatively easy to assay and can suggest mechanisms associated with sperm competition, the complex nature of differential paternity can often complicate the identification of single gene effects. Moreover, the precise mechanisms through which candidate genes exert their role remains unknown in the absence of a more fine-grained phenotypic categorization (see previous section). These first two challenges are examined next.

### Quantifying Heritable Genetic Variation in Sperm Competitive Ability

The amount of heritable, *i.e.*, additive, genetic variation (V_A_) underlying sperm-related traits presumably linked to sperm competitive ability has been performed in multiple species from different taxa, finding extensive support to the presence of abundant V_A_ associated with such traits (Woolley, 1971; Mossman et al., 2009; Dobler and Hosken, 2010; Minoretti et al., 2013). In contrast, equivalent studies, but focused on sperm competitive ability, are much more limited and usually confined to arthropods. These studies measured paternity contribution scores finding either nonsignificant or very low heritability (*h^2^*) values (Gilchrist and Partridge, 1997; Hughes, 1997; Friberg et al., 2005; Konior et al., 2005; Hosken et al., 2008; Tregenza et al., 2009; Dowling et al., 2010). These low *h^2^* values are compatible with intense directional selection depleting V_A_, coupled or not with stabilizing selection, although they could be also explained by vast non-additive effects, which are known to dramatically affect the outcome of sperm competition (Hughes, 1997). These non-additive effects would contribute to a large residual variance, *i.e.*, the one that encapsulates both non-additive (*i.e.*, dominant and epistatic) genetic and environmental effects, thus explaining low *h^2^* values (Houle, 1992). These effects derive from genotypic differences associated with male × male interactions (Clark et al., 2000), with male × female and male × male × female interactions (Clark and Begun, 1998; Clark et al., 1999; Birkhead et al., 2004; Bjork et al., 2007; Chow et al., 2010), and with interactions between the nuclear and mitochondrial genomes (Yee et al., 2013). The substantial impact of non-additive effects to the systematic underestimation of V_A_ underlying sperm competitive ability was confirmed in a review of 44 studies that included 19 species in which V_A_ was calculated for different classes of sperm-related traits, which also differed in how they are affected by non-additive effects (Simmons and Moore, 2008). One class—fertilization traits—that comprised sperm competitive success showed the largest variance around the average estimate of V_A_.

At least two other types of studies more directly gauging the magnitude of V_A_ have been performed. The first type corresponds to experimental evolution studies in which the intensity of sexual selection and sexual conflict is modified by modulating the operational sex ratio or enforcing extreme monogamy. In *D. melanogaster*, the detection of a response to selection in relation to paternity contribution scores has been equivocal and often dependent on the experimental scheme followed (Rice, 1998; Bjork et al., 2007; Jiang et al., 2011; Nandy et al., 2013; Wensing et al., 2017). In contrast, equivalent studies in the horned beetle *Onthophagus taurus* and the house mice *Mus musculus* have provided wide support to the evolution of sperm competitive ability under induced selection regimes (Simmons and Garcia-Gonzalez, 2008; Firman and Simmons, 2011). Further, genetic variant association studies in *D. melanogaster* (see below) have provided strong evidence of heritable V_A_ underlying paternity contribution scores. Collectively, these results highlight the challenge of detecting and measuring V_A_ and that there is potentially more V_A_ underlying sperm competitive ability than suggested by *h^2^* studies and some experimental evolution studies.

### Identifying Candidate Genes

QTL-mapping studies have been performed to identify genomic regions that can account for a significant fraction of the variation in sperm competitive ability. In *Drosophila*, QTL mapping failed to reveal any locus of major effects associated with paternity contribution scores (Hughes, 1997; Lawniczak and Begun, 2005; Hughes and Leips, 2006), pointing to a multifactorial genetic basis, which is in line with what has been found for many other sexually selected traits (Chenoweth and McGuigan, 2010). Nevertheless, in two sister species of *Peromyscus* mice (see below), QTL mapping was successful in identifying a genomic region that harbors a gene of large effect, *protein kinase cAMP-dependent regulatory type I alpha* or *Prkar1a* (Fisher et al., 2016). The relative prevalence of these two very different emerging architectures associated with sperm competitive ability remains to be determined.

Other *Drosophila* studies evaluating the degree of association between naturally occurring V_A_ and sperm competitive ability have uncovered very promising candidate genes. A genome-wide-SNP-association study pointed to 33 genes as candidates to influence sperm competitive ability through their effects in females, finding an unexpected overrepresentation of neuronal genes (Chow et al., 2013). Previously, association studies performed in this same species focused on particular male ACP-encoding genes, as the site of gene expression was suggestive of their role in sperm competition (Clark et al., 1995; Fiumera et al., 2005; Fiumera et al., 2007; Wong et al., 2008; Greenspan and Clark, 2011; Zhang et al., 2013). In all these association studies, isogenic lines were used trying to minimize genetic background effects to facilitate the detection of such associations. Nevertheless, some caveats in these studies should be noted. First, these studies yielded slightly different results for some genes when exposed to different population-specific genomic backgrounds (Clark et al., 1995; Fiumera et al., 2005; Fiumera et al., 2007). Second, false positives, even after adjusting for multiple tests, cannot be eliminated in this type of studies, and therefore, different associations might be found depending on the level of stringency utilized. Third, association studies do not provide evidence for causation, and any presumed significant association should be validated by follow-up functional assays such as those involving perturbation of gene activity. This has been done only in some cases, including the ACP-encoding genes *Acp36DE* and *Acp62F*, and the neural-related genes *paralytic* (*para*) and *Rab2* (Mueller et al., 2008; Avila and Wolfner, 2009; Chow et al., 2013).

A series of different approaches, rather than strict genetic variant association studies, have also been used. Some studies have directly used particular functional and evolutionary gene attributes to define candidates that can influence reproductive fitness through sperm competition. This is the case of the *D. melanogaster* specific multigene *sperm-specific dynein intermediate chain* or *Sdic* (Nurminsky et al., 1998), which shows a peak of expression in testes and is a defective version of another gene that encodes a cytoplasmic axonemal protein, which was suggestive of a role in sperm motility. These hallmarks prompted the evaluation of its relationship with sperm competitive ability in competitive setting upon perturbation of its function (Yeh et al., 2012). Another example is the rodent gene *cation channel sperm associated 1* (*Catsper1*), which encodes a protein that is known to impact sperm motility, exhibits a lineage-specific signature consistent with the action of positive selection, and for which there is a correlation between shorter forms of the encoded protein and lineages with increasing sperm competition levels (Vicens et al., 2014).

This class of studies has also been implemented at a genome level. A comparative analysis between the nematode *Caenorhabditis briggsae* and its selfing sibling species *Caenorhabditis nigoni* revealed differential gene content with an enrichment of male-biased genes in expression unique to the outcrossing *C. nigoni* species. One missing genetic factor in selfing species was the *male secreted short* (*mss*) tandemly arranged multigene family, which encodes a sperm membrane protein and is present in all outcrossing species in a variable number of paralogs, becoming primary candidate for subsequent perturbation experiments (Yin et al., 2018). Further, a different comparative analysis performed across the *Drosophila* phylogeny identified six genes whose encoded proteins were characterized by co-varying in their rates of evolution with other proteins known to interact with the *sex peptide* (*SP* or *Acp70A*) protein (Findlay et al., 2014). The SP protein binds sperm stored in the female and has been shown to be relevant for sperm competitive ability (see next section). Lastly, a special class of genome-wide searches corresponds to high-throughput expression studies such as those that examine the presence and abundance of proteins in sperm and seminal fluid (Ramm et al., 2015; Zhou et al., 2015; Vicens et al., 2017; Dosselli et al., 2019; Rowe et al., 2019). For example, in *M. musculus*, by tuning the competition risk among males, it was possible to identify seminal fluid proteins that exhibit changes in relative abundance (Ramm et al., 2015). Likewise, in *A. colombica*, seven seminal fluid proteins that are known to be degraded by exposure to spermathecal fluid while in female storage were identified, prompting further experiments (Dosselli et al., 2019).

### Competitive Settings and Functional Verification

To reliably identify genetic factors linked to sperm competition phenotypes, it is necessary to test for evidence of phenotypic variation in competitive settings as well as to implement functional tests that can account for confounding factors and strengthen the causal link between the candidate gene and sperm competition phenotypes. Relative to the first requisite, many candidate genes await to be tested in appropriate competitive conditions. That is the case of genes such as those in *D. melanogaster lost boys* (*lobo*) and *Diuretic hormone 44* (*Dh44*), which have been shown to ultimately alter sperm storage (Yang et al., 2011; Lee et al., 2015). The genes *protamine 1* and *2* (*Prm1* and *Prm2*, respectively) are additional examples. In the case of *Prm1*, a sequence evolution study across mammalian clades found that the intensity of sperm competition was significantly correlated with arginine content in the encoded product, being particularly the case in rodents and cetaceans. Interestingly, high arginine content was found to be correlated with slimmer sperm heads (Luke et al., 2016). In the case of *Prm2*, expression differences across eight closely related species in the genus *Mus* also exhibit strong association with different intensities of sperm competition operating on particular species, nucleotide changes in the promoter sequence, and differences in sperm head morphology (Martin-Coello et al., 2009a; Martin-Coello et al., 2009b; Luke et al., 2014). *In vitro* competitive assays should assess whether differential hydrodynamic properties due to sperm head morphology divergence result in varying sperm swimming and ultimately competitive ability.

The need of testing candidate genes in sperm competitive settings is even more relevant for those delineated based on generic functional or evolutionary gene attributes. For example, mRNA or proteomic expression surveys involving reproductive tissues, particularly those for which there is evidence that expression can result more closely in sexual antagonism, might be misleading regarding to implication in sperm competition (Edward et al., 2014). First, not all the genes preferentially expressed in tissues such as the male accessory gland proteins and the female spermatheca show signatures of sexual antagonism (Innocenti and Morrow, 2010), and second, when some of those genes (*e.g.*, *Acp63F*, *Acp95EF*, and *Acp98A*) are tested in SNP-association studies in relation to sperm competitive phenotypes, no effect was found (Fiumera et al., 2007).

Ultimately, functional tests of differential sperm competitive ability are also essential. This has been done for example with many *D. melanogaster* ACP-encoding genes, due to their proven effect on paternity contribution scores in association studies (Clark et al., 1995; Fiumera et al., 2005; Fiumera et al., 2007). An obvious possibility has been to silence partially or entirely the activity of gene candidate to confirm its implication in sperm competition. In model organisms, engineering mutants is routine either through already established collections of genetically modified strains for many of the genes in the genome (Spradling et al., 1999; Rual et al., 2004; Heigwer et al., 2018) or the generation of new ones using more recently developed methodologies such as CRISPR-Cas9. Importantly, CRISPR-Cas9 has also democratized the possibility to perform perturbing assays beyond model organisms (Mendoza and Trinh, 2018). Additionally, CRISPR-Cas9 offers the possibility to simultaneously target multiple genes (Zhang et al., 2018) and therefore to explore gene interactions, improving the functional annotation of the genes involved (*e.g.*, sperm storage or retention in a particular female organ). Finally, functional tests other than the perturbation of gene activity are becoming increasingly implemented, particularly when engineering mutations are not an obvious choice. In *A. colombica*, whether particular seminal protein proteases play a key role in the sperm incapacitation observed was determined by exposing mixtures of seminal fluid from competing males to different cocktails of protease inhibitors (Dosselli et al., 2019).

## Genes Involved

We focused on those genes for which there is an unequivocal link to sperm competition phenotypes as tested in competitive setting and for which functional assays have been done. Thirty-three genes in fruit flies, 2 in ants, 8 in rodents, and 2 in nematodes fit with our outlined criteria (**Table 1**). This gene set includes from those that exhibit phenotypic effects in competitive settings but not in settings without competition, *e.g.*, no detectable fertility decrease in single matings or impaired sperm motility (Sutton et al., 2008; Yeh et al., 2012; Yin et al., 2018), to others that already exhibit some phenotypic effects denoting limited reproductive success in no competitive settings (Sitnik et al., 2014). Some key examples arranged by broad functional themes are examined next, with emphasis on genes identified outside the taxon most widely studied (*i.e.*, *Drosophila*). This categorization is, in many cases, limited by the number of functional studies so far undertaken. It is therefore important to note that, given the pleiotropic nature of some genes, alternative arrangements could surface depending on new information.

**Table 1:** Genes involved in sperm competition.

Organism	Gene	Experimental design*	Paternity contribution score influenced	Functional mechanism^II^	Tissue and/or cellular localization^†^	Reference
Flies *(Drosophila)*	*Abd-B*	Gp, Pc	*P1*	Egg laying and remating; sperm storage; gonadal development	Accessory gland	(Gligorov et al., 2013)
	*Acp29AB*	Ga,Gp, Pc	*P1, P2*	Sperm retention; sperm storage	Accessory gland	(Clark et al., 1995; Fiumera et al., 2005; Wong et al., 2008)
	*Acp36DE*	Ga, Gp, Pc	*P1*	Sperm storage; mating plug formation; postmating female receptivity	Accessory gland	(Clark et al., 1995; Avila and Wolfner, 2009; Avila and Wolfner, 2017)
	*Acp62F*	Ga, Gp, Pc	*P1, P2*	Unknown (affects processing of ovulin)	Accessory gland	(Fiumera et al., 2007; Mueller et al., 2008)
	*btsz*	Gp, Pc	*P1*	Sensory	Neuronal	(Chen et al., 2019)
	*caup*	Gp, Pc	*P1*	Sensory	Neuronal	(Chen et al., 2019)
	*cdc14*	Gp, Pc	*P1, P2*	Unknown; microtubule organization	Testes	(Neitzel et al., 2018)
	*CG6864*	Gp, Pc	*P2*	Unknown (sperm storage, displacement); spermatogenesis	Testes	(Civetta and Finn, 2014)
	*CG9997*	Gp, Pc	*P2*	Egg laying, sperm release; postmating female receptivity	Accessory gland	(Ram and Wolfner, 2007b; Ram and Wolfner, 2009; Castillo and Moyle, 2014)
	*CG14891*	Gp, Pc	*P2*	Unknown (sperm viability, function)	Testes	(Civetta and Finn, 2014)
	*CG17575*	Gp, Pc	*P1*	Sperm retention; postmating female receptivity	Accessory gland	(Ram and Wolfner, 2007b; Avila and Wolfner, 2009)
	*CG31872*	Gp, Pc	*P1*	Unknown	Ubiquitous	(Chen et al., 2019)
	*CG32834*	Gp, Pc	*P1*	Unknown	Ubiquitous	(Chen et al., 2019)
	*Ddr*	Gp, Pc	*P1*	Unknown	Ubiquitous	(Chen et al., 2019)
	*Dnah3*	Gp, Pc	*P2*	Sperm swimming (cilium movement)	Chordotonal neurons, sperm	(Karak et al., 2015)
	*Dnai2*	Gp, Pc	*P2*	Sperm swimming (cilium movement)	Chordotonal neurons, sperm	(Karak et al., 2015)
	*Est-6*	Gp, Pc	*na*	Sperm release, sperm storage; postmating female receptivity; pheromone biosynthesis; courtship behavior; ovulation; oviposition	Ejaculatory duct	(Gilbert, 1981;Clark et al., 1995;Fiumera et al., 2007)
	*hid*	Gp, Pc	*P1*	Sensory	Neuronal	(Chen et al., 2019)
	*lectin-46Ca*	Gp, Pc	*P1*	Sperm release; postmating female receptivity	Accessory Gland	(Ram and Wolfner, 2007b; Avila and Wolfner, 2009)
	*lectin-46Cb*	Gp, Pc	*P1*	Sperm release; postmating female receptivity	Accessory Gland	(Ram and Wolfner, 2007b; Avila and Wolfner, 2009)
	*mir-973*	Gp, Pc	*P2*	Sperm morphology, female remating	Testes	(Lu et al., 2018)
	*mir-978*	Gp, Pc	*P1, P2*	Sperm morphology	Testes	(Lu et al., 2018)
	*mir-983*	Gp, Pc	*P2*	Sperm morphology, female remating	Testes	(Lu et al., 2018)
	*Msp300*	Gp, Pc	*P1*	Sensory	Neuronal	(Chen et al., 2019)
	*Nep2*	Gp, Pc	*P1*	Sperm release	Brain, testes	(Sitnik et al., 2014)
	*Ovulin, Acp26Aa*	Ga, Gp, Pc	*P1*	Postmating behavior; postmating oviposition	Accessory gland	(Clark et al., 1995; Rubinstein and Wolfner, 2013)
	*para*	Ga, Gp, Pc	*P1*	Sensory; male courtship behavior		(Chow et al., 2013)
	*Pkd2*	Gp, Pc	*P2*	Sperm storage, sperm release; sperm swimming (flagellated sperm motility); spermatogenesis	Sperm	(Watnick et al., 2003; Kottgen et al., 2011)
	*Rab2*	Ga, Gp, Pc	*P1*	Sensory; neural cell body	Neuronal	(Chow et al., 2013)
	*Rim*	Ga, Gp, Pc	*P1*	Sensory; neurotransmitter secretion; synaptic vesicle	Neuronal	(Chow et al., 2013; Chen et al., 2019)
	*Sdic*	Gp, Pc, St	*P2*	Sperm displacement; microtubule-based movement	Testes	(Yeh et al., 2012; Jayaswal et al., 2018)
	*SP, Acp70A*	Ga, Gp, Pc	*P1, P2*	Postmating oviposition, postmating female receptivity; sperm release	Accessory gland	(Avila et al., 2010; Castillo and Moyle, 2014)
	*SPR*	Ga, Gp, Pc	*P1, P2*	Postmating female receptivity (neuronal sensory); postmating oviposition	FRT	(Chow et al., 2010; Smith et al., 2017)
Worms *(Caenorhabditis)*	*comp-1*	Gp, Pc, Sp	*P1, P2*	Sperm storage	Sperm pseudopod	(Hansen et al., 2015)
	*mss*	Gp, Pc, Sp	*P1, P2*	Unknown	Sperm membrane	(Yin et al., 2018)
Mice *(Peromyscus, Mus)*	*Acr*	Gp, Sp	*na*	Sperm development, acrosomal vesicle; acrosome reaction; fertility (binding of sperm to zona pellucida)	Sperm	(Adham et al., 1997; Nayernia et al., 2003)
	*Catsper1*	Gp, Sp	*na*	Spermatogenesis; sperm capacitation; fertility (fusion of sperm to egg plasma); sperm swimming (flagellated sperm motility, cilium beat frequency)	Sperm	(Ren et al., 2001; Zeng et al., 2013; Vicens et al., 2014)
	*CEACAM10*	Gp, Msm	*na*	Sperm swimming (flagellated sperm motility)	Seminal vesicle	(Li et al., 2005; Ramm et al., 2015)
	*CRISP1*	Gp, Sp	*na*	Sperm development and Fertility; cytoplasmic vesicle	Sperm head	(Da Ros et al., 2008)
	*Pate4, Svs7*	Gp, Msm	*na*	Sperm retention/release; acrosomal vesicle	Seminal vesicle	(Ramm et al., 2015; Noda et al., 2018)
	*Pkdrej*	Gp, Pc, Sp	*na*	Sperm development and fertility (acrosome reaction)	Anterior sperm head	(Sutton et al., 2008)
	*Prkar1a*	Sp	*na*	Sperm swimming (axoneme), morphology	Sperm midpiece	(Fisher et al., 2016)
	*Svs2*	Gp, Msm	*na*	Sperm viability; sperm development, fertility; acrosomal vesicle; sperm capacitation	Seminal vesicle	(Kawano et al., 2014; Araki et al., 2015; Ramm et al., 2015)
Ants *(Atta)*	*Easter*	Pp, Sp	*na*	Sperm viability	Sperm	(Dosselli et al., 2019)
	*Snake*	Pp, Sp	*na*	Sperm viability	Sperm	(Dosselli et al., 2019)

*Ga, gene association study; Gp, gene perturbation study; Pc, paternity contribution test; Sp, sperm-tracking test; Msm, mating system manipulation; Pp, protein perturbation.

^II^As it relates to sperm competition based on primary literature and AmiGO2 (http://amigo.geneontology.org/amigo/search/bioentity).

^†^As in FlyBase (http://flybase.org/) in the case of D. melanogaster. FRT, female reproductive tract.

### Sperm Morphology and Swimming

Sperm are the most diverse cell type known, and changes in length and shape can influence sperm motility and therefore sperm competitive ability (Pitnick et al., 2009; Pizzari and Parker, 2009; Lupold et al., 2016). One example of a gene influencing sperm morphology and its movement is *Prkar1a*. The deer mice (*Peromyscus maniculatus*) and the old-field mice (*Peromyscus polionotus*) exhibit highly divergent mating systems, the first being extremely promiscuous and the second being strictly monogamous. This interspecific difference influences how sexual selection operates on these species, being reflected in important differences for traits such as testis size and sperm velocity, known to be associated with sperm competition. Interspecies F2 hybrids heterozygous for the promiscuous *P. maniculatus Prkar1A* allele have longer sperm section called midpiece. The length of the midpiece has been shown to correlate with swimming velocity in *in vitro* assays between competing sperm, enhancing the probability of siring progeny (Fisher et al., 2016).

In the case of other genes, either morphology or motility are affected, impacting sperm competition. In *D. melanogaster*, a study on six noncoding genes that regulate gene expression post-transcriptionally (miRNA genes) found that three were functionally related primarily to male reproduction, including sperm competition (Lu et al., 2018). Interestingly, *mir-978* knockout exhibited a significant effect in both offense and defense settings, while the knockouts of *mir-973* and *mir-983* did so only in the former. The knockouts of all these genes had significantly longer sperm than controls. These miRNA genes are also widely pleiotropic, impacting other components of fitness (*e.g.*, viability). Further, sperm movement can also be influenced by the presence of seminal fluid secretions, as it has been shown by treatment of fresh sperm with or without the *M. musculus* protein carcinoembryonic antigen-related cell adhesion molecule 10 or CEACAM10 (Li et al., 2005). This protein is secreted by the seminal vesicle, a male accessory sexual gland. Sperm movement was significantly augmented in samples incubated with CEACAM10 (Li et al., 2005). A proteome analysis in which mice were exposed to high *versus* low risk of sperm competition singled out CEACAM10 as a protein significantly more abundant in the high-competition treatment group (Ramm et al., 2009).

### Sperm Storage

Sperm entry and proper placement in storage (*e.g.*, near the exit for fertilization) are critical for competitiveness and fertilization success. One of the best functionally characterized proteins is Acp36DE in *Drosophila*, which mediates the elongation of the uterus after mating. The Acp36DE protein is a component of the anterior mating plug, helping to corral sperm close to the opening of sperm storage organs (Bertram et al., 1996; Avila and Wolfner, 2009; Avila et al., 2015). The absence of the Acp36DE protein from the ejaculate results in lower number of sperm stored and decreased fertility (Neubaum and Wolfner, 1999; Qazi and Wolfner, 2003). Importantly, perturbation of this gene influences sperm competitiveness (Castillo and Moyle, 2014). Sequence variation at this gene was found to influence male’s sperm defensive ability when testing isogenic lines derived from a North Carolina population but not when using lines derived from a Pennsylvania population (Clark et al., 1995; Fiumera et al., 2005).

Hermaphroditic *C. elegans* are self-fertile as they undergo spermatogenesis prior to oogenesis. These types of individuals can receive sperm and produce outcrossed progeny, creating opportunities for sperm competition. The gene c*omp-1* is expressed in the germ line, but it is not involved in sperm development. Instead, *comp-1* is required for proper migration and localization of sperm within the spermathecae (Hansen et al., 2015). Sperm from knockout males show unusual dynamics although no basal motility defects (*e.g.*, in directionality). Unlike wild-type sperm, sperm from knockout males are largely absent from the spermathecae, which ultimately hampers their potential to fertilize the oocytes. This localization defect is context-dependent, suggesting that *comp-1* is ultimately involved in coordinating environmental signals that influence successful migration and localization of the sperm in the reproductive tract. Overall, *comp-1-*deficient male sperm perform poorly both in offensive and defensive competitive settings.

### Sperm Retention and Release

Following transfer and entry of sperm into female storage organs, proper sperm release is often critical to guarantee efficient fertilization. In *Drosophila*, the ACP-encoding gene *SP* is highly pleiotropic, eliciting a wide variety of physiological responses such as increased aggression and repressed immune system (Sirot, 2019). A few responses are more directly linked to sperm competitiveness outcome. Specifically, stimulation of ovulation and egg-laying and inhibition of remating, as well as proper sperm release from the female storage organs, results in increased P1 when *SP* is knockout (Avila et al., 2010). *SP* gene perturbation significantly reduce a male’s ability to father progeny when second to mate (P2) (Castillo and Moyle, 2014) making *SP* an interesting example of a possible trade-off in that functional variants of the gene result in opposite performances in terms of P1 and P2 (Avila et al., 2010; Castillo and Moyle, 2014). Further, in rodents, the seminal vesicle of these species group secretes a family of proteins with a potential role in sperm competition (Ramm et al., 2009; Ramm et al., 2015). Under conditions of high sperm competition, the mouse seminal vesicle secretion protein 7 (SVS7) was shown to increase in concentration (Ramm et al., 2015). Knockouts of the SVS7-encoding gene form a smaller copulatory plug in the female vagina causing sperm leakage, demonstrating a functional role of SVS7 in sperm retention (Noda et al., 2018).

Clearly, females are far from being just passive participants in sperm competitive outcomes as they might selectively use sperm from particular males, a mechanism referred to as cryptic female choice (Eberhard, 1996). An obvious way in which this can happen is by impacting sperm release from the storage organs. The receptor of SP in the *D. melanogaster* female, which is encoded by the *sex peptide receptor* (*SPR*) gene, mediates the effects of *SP* on P2 as evidence shows that the effect of particular male *SP* alleles are dependent on the female *SPR* allele (Chow et al., 2010). The use of females with a genetically modified version of *SPR* has subsequently shown that *SPR* also influences females remating and fecundity (Smith et al., 2017).

### Female Neuronal Effects on Sperm Storage

Multiply mated females can also bias competitive outcomes through their nervous system, which is known to be important for proper sperm storage (Arthur et al., 1998). For example, a neuron-specific sensory knockdown in females of the *D. melanogaster* gene *Rab3 interacting molecule* (*Rim*), a gene that mediates neurotransmitter secretion, lowers males P1 (Chow et al., 2013; Chen et al., 2019). Similarly in *D. melanogaster*, the gene *Caupolican* (*caup*), which is generally involved in neuronal development, has been shown to affect males P1 when knockdown in female’s octopaminergic neurons, and both P1 and P2 but not overall fertility when knockdown across the whole nervous system of the female (Chen et al., 2019). Overall, both octopaminergic *Tdc2*+ and proprioceptive *ppk*+ neurons, which innervate the female reproductive tract, have been found to play roles in competitive settings. Although the precise mechanisms whereby this type of neural-related genes affects sperm storage are still unknown, it has been proposed that this could happen by how the female nervous system integrates signals from courtship and ejaculates (Chen et al., 2019).

### Sperm Displacement From Storage

Once in storage, sperm faces challenges from competing sperm that can simply physically eject or displace them to positions in storage that are further away from accessible eggs for fertilization. *Sdic* is largely expressed in testes, showing an effect on P2, but not on P1, when knockout. Male knockouts of *Sdic* did not show impaired basal sperm motility and functionality, but visual tracking of sperm revealed that the reproductive bias in competitive settings resulted from a less effective displacement of previous resident male sperm from the female storage organs (Jayaswal et al., 2018).

### Sperm Viability

During storage, sperm faces challenges from both the female environment and potentially other incoming ejaculate that can severely affect their viability. Knockouts of the mouse gene *seminal vesicle protein 2* (*SVS2*) have been shown to affect the formation of the copulatory plug and fertility. A series of *in vivo* tests ruled out different parameters that might affect the *SVS2* knockout fertility. Instead, in using artificial insemination with silicon as a substitute for the copulatory plug, and both electron microscopy and sperm cell stain assays, it was shown that the lack of SVS2 causes sperm fracture and death (Kawano et al., 2014). Crucially, in a proteomics survey that exposed males to conditions of high and low risks of sperm competition, mice increased the protein SVS2 production under conditions of high risk (Ramm et al., 2015). Additionally, *SVS2* has also been shown to influence sperm capacitation (Araki et al., 2015). These studies identify *SVS2* as a gene that influences sperm competition through its effect on sperm viability while underscoring the pleiotropic nature of some sperm competition genes.

A proteomics survey in *A. colombica* identified seminal fluid proteins that are degraded by exposure to spermathecal fluid while in female storage (Dosselli et al., 2019). By using an *in vitro* design in which seminal fluids from focal and rival males were exposed to spermathecal fluids, the authors established that exposure of seminal fluids to spermathecal fluids indiscriminately preserves sperm survival. Moreover, using different protease inhibitor cocktails, the authors show that only the inhibitors that degrade the serine proteases encoded by the genes *Snake* and *Easter* diminish sperm mortality when exposed to rival seminal fluid. Importantly, this study highlights the role of females in impairing sperm viability and therefore sperm competitive ability (Dosselli et al., 2019).

### Egg Laying and Remating

Among internal fertilizers, the transfer of male seminal proteins triggers different female physiological responses including an increase in ovulation and egg-laying (Robertson, 2007; Avila et al., 2011). Males capable of triggering drastic increases in ovulation shortly after mating are expected to benefit by increasing their chances to fertilize eggs when facing post-mating competition. Often, increases in egg-laying go hand in hand with refractoriness to remate. In *D. melanogaster*, nucleotide polymorphism in the gene *ovulin* (*Acp26Aa*) is associated with variable P1 (Clark et al., 1995). One-way *ovulin* polymorphism could mediate sperm competitiveness is if this variation is correlated with variable egg-laying. Knockout males have shown how *ovulin* influences egg-laying through neural signaling on the oviduct musculature of mated females (Rubinstein and Wolfner, 2013).

The relevance of the seminal proteins in the post-mating response in the females can be tracked back to its proper production and processing. The homeotic gene *abdominal-B* (*Abd-B*) of *D. melanogaster* is well known for contributing to specify the identity of some of the abdominal segments of the fly, including gonads and the regulation of proper stem cell architecture and testes development (Sanchez-Herrero et al., 1985; Papagiannouli et al., 2014). *Abd-B* is also expressed in a differentiated set of cells (the so-called secondary cells) within the male accessory glands, impacting on the functional properties of four ACPs. This in turn affects SP and ultimately the maintenance of the post-mating response (Gligorov et al., 2013). Underexpression of *Abd-B* in the secondary cells affects their development and expression profile, resulting in anomalous triggering of long-term egg laying and suppressing female receptivity to remate, thus affecting sperm competitiveness (Gligorov et al., 2013).

### Sperm Development and Fertility

Competitive sperm must properly develop to become fully functional and fertilize an egg. Non-properly executed key events prior to fertilization of the egg can diminish sperm competitive ability. These key events are sperm capacitation, *i.e.*, the reprogramming that the sperm undergo to become functional, and acrosome reaction, *i.e.*, the sequence of molecular steps required for the sperm to penetrate the zona pellucida and fuse with the oocyte membrane. In mice, the absence of the male germ line protein acrosin prepropeptide *(Acr)*, a protease that is activated during the acrosome reaction, does not significantly affect male spermatogenesis or fertility. However, mice knockouts for *Acr* have a disadvantage in the presence of other sperm in sperm competition assays (Adham et al., 1997; Nayernia et al., 2003). Another example in mice is the gene cysteine-rich secretory protein 1 (*Crisp1*)*. Crisp1* is a member of an evolutionary conserved multigene family that encodes a protein relevant both during capacitation and gamete fusion. Knockout males were fertile but showed reduced sperm ability to penetrate both normal and zona pellucida-free oocytes as well as the sperm fusion ability in competition assays (Da Ros et al., 2008).

## Sperm Competition and Speciation

When females mate with both conspecific (same species) and heterospecific (different species) males, the conspecific male sires the majority of progeny regardless of the mating order. This conspecific sperm precedence (CSP) can act as a postmating prezygotic reproductive isolation barrier between closely related species, which has been shown to be a common form of isolation in a wide variety of species (Price, 1997; Howard et al., 1998; Matsubayashi and Katakura, 2009; Tyler et al., 2013). Nevertheless, there have not been many studies addressing the rate of evolution of CSP. Differences in rates of evolution among pre- and postmating forms of isolation can help identify the relevance of such mechanisms as barriers to gene flow during early stages of speciation. A recent survey among species of the *D. melanogaster* subgroup identified CSP as the second fastest evolving reproductive isolation mechanisms after premating isolation (Turissini et al., 2018). Moreover, postcopulatory sexual selection can accelerate divergence between reproductive traits, thus phenotypic characterization of sperm competition in related species can help identify the most relevant molecular processes that can contribute to the buildup of reproductive isolation. For example, detailed characterization of sperm competition phenotypes and sperm dynamics using differentially labeled sperm in transgenics of *D. simulans* and *Drosophila mauritiana* has revealed many commonalities with *D. melanogaster* but also important differences. Unlike *D. simulans* and *D. mauritiana*, *D. melanogaster* females store sperm preferentially in the seminal receptacle relative to the spermathecae and *D. mauritiana* females eject sperm sooner than *D. simulans* and *D. melanogaster* females (Manier et al., 2013a). Moreover, another investigation using sperm-transgenic lines in competitive settings provided strong support for sperm displacement, and to less extent, to sperm ejection and fertilization bias, as causal speciation phenotypes (Manier et al., 2013b).

Unfortunately, progress in our understanding of the mechanistics of sperm competition and CSP at the interspecific level has not been matched by biochemical studies, as it has not been the relative weight between biochemical *versus* morphological and behavioral incompatibilities (Markow et al., 2007). A number of biochemical processes are known to modulate the physiological status of sperm in storage and its competency in fertilization (Markow et al., 2007). Whether divergence between species in postcopulatory–prezygotic biochemical interactions can impair sperm fertilization success, thus contributing to reproductive isolation, remains underexplored. Detail characterization of sperm competition and CSP, both mechanistically and biochemically, among different groups of closely related species, is required.

An intriguing question to be addressed is whether genes that influence intraspecific sperm competition (ISC) also contribute to CSP. A common genetic basis for ISC and a postmating reproductive isolation phenotype like CSP would provide grounds for an uncontested connection between sexual selection and speciation (Panhuis et al., 2001; Ritchie, 2007; Safran et al., 2013; Seddon et al., 2013; Servedio and Burger, 2014). Up to date, very few studies have attempted to map the genetic basis underlying CSP (Civetta et al., 2002; Britch et al., 2007; Fishman et al., 2008; Levesque et al., 2010), and even fewer have followed up on possible candidate genes within mapped loci. One follow-up molecular evolution study focusing on five candidate ACP-encoding genes within a previously mapped CSP QTL (Civetta et al., 2002) singled out *Acp53C14c* as the only candidate gene of interest for future gene-targeting studies attempting to validate its role in CSP between *D. simulans* and *Drosophila sechellia* (Civetta and Reimer, 2014). Further, two studies have directly examined possible gene-specific connections between ISC and CSP. In one study, *D. melanogaster* lines carrying non-functional copies for ACP-encoding genes were tested against *D. melanogaster* or *D. simulans* males. Two genes, *Acp36DE* and *CG9997*, were found to contribute to ISC and CSP, while *SP* affected ISC but not CSP (Castillo and Moyle, 2014). Further, genetically modified versions of previously identified CSP candidate genes (Levesque et al., 2010) were tested in competitive assays in *D. melanogaster*, with two of them, *CG14891* and *CG6864*, influencing ISC outcomes (Civetta and Finn, 2014). Systematic surveys using perturbation of the activity of additional candidate genes should allow us to determine the extent of a shared genetic basis between ISC and barriers to gene flow, like CSP, that contribute to speciation or the maintenance of species boundaries.

## Conclusions and Future Directions

Our understanding on the genetic basis of sperm competition has increased considerably since Geoffrey Parker proposed its importance (Parker, 1970). Among the genetic factors that we can safely deem as sperm competition genes, we observe a remarkable breadth of biological roles, a marked pleiotropic nature in many of them, and multiple examples of genes not expressed in testes or the sperm (∼56% in **Table 1**). Importantly, staggering evidence supports that the fertilization bias that results from post-copulatory male–male competition can be exerted by genes whose effects not only manifest through the males but also through the females (Chen et al., 2019). Nevertheless, this gene list is still limited, and biased toward *D. melanogaster* and research foci such as the characterization of the male accessory glands of this species. With the systematic implementation of experimental competitive settings and the generalization of the use of functional assays that allow to monitor gene activity, the properties of the resulting molecules, and sperm features and behavior, we envisage an increased ability in identifying genes that bias sperm competitive outcomes as well as a more refined phenotypic and biochemical categorization of sperm competition across a variety of species from distantly related taxa.

Although some validated genes exhibit a clear link to sperm competition phenotypes, the underlying genetic variants that cause that phenotypic variation in natural populations remain mostly unidentified. Equally important, in spite of having examples of SNPs and small indels within the open reading frame of genes that influence such phenotypes (Fiumera et al., 2007; Greenspan and Clark, 2011), it is still unclear the extent to which other types of genetic changes contribute to differential sperm competitive ability. For example, in the case of the gene *Prkar1a* (Fisher et al., 2016), a difference in expression level but not in the amino acid sequence of the encoded protein was documented. This change in expression was presumably the result of genetic change in the regulatory region of the gene. In other cases, structural variants, which have been largely omitted in reference genome assemblies and surveys of V_A_ (Huddleston and Eichler, 2016; Chakraborty et al., 2018), might be responsible for such differences in mRNA abundance. For example, *Sdic* and *mss* are tandemly arranged multigene families, pointing to the possibility that their copy number variation may be responsible for differences in sperm competitive ability. To sum up, surveys of V_A_ at the sequence, structure, and function levels will have to be implemented for genes lacking such data, which will help gauge the relationship between sperm competition phenotypes and different types of V_A_.

Studies incorporating naturally occurring variation will also allow us to address questions regarding the role of different types of selection and population structure in driving the molecular evolution of already validated sperm competition genes both within and between closely related species. These molecular diversity studies will have to incorporate particularities of the genes involved. For example, validated genes with sex-limited expression are expected to be more prone to experience intra-locus sexual conflict, which would limit adaptive evolution (Mank, 2017). The magnitude of the sex-biased effects should be considered as it affects the rate of evolution (Harrison et al., 2015; Dapper and Wade, 2016), and so must be the intensity of sperm competition as well as the context in which selection might operate, *e.g.*, haploid *versus* diploid stage for sperm expressed genes, which is correlated for some genes with late *versus* early expression during spermatogenesis (Dapper and Wade, 2016). Likewise, the pleiotropic nature of some of these genes, *e.g.*, ACP-encoding genes through their effects on female fitness, links intra- and inter-locus sexual conflicts, offering additional ways in which the strength of selection might be tuned (Dapper and Wade, 2016).

Lastly, extensive comparative genomic studies have revealed that a sizable fraction of model organism genes expressed in—for example, the sperm, have an ortholog in humans (Okabe et al., 1998; Karr, 2007), with some of these genes showing extremely similar mutant phenotypes associated with deficient fertility (Kusz-Zamelczyk et al., 2013; Yu et al., 2015). It is entirely conceivable that some sperm competition genes can help identify human orthologs that play a role in human pathologies associated with variable fertility, which will foster further investigation about the link between sperm competition genes and male fertility in humans.

## Author Contributions

Both authors have made equal contributions to the work, and approved it for publication.

## Funding

This work was supported by grants from the Natural Sciences and Engineering Research Council of Canada NSERC-RGPIN-2017-04599 to AC and the National Science Foundation NSF-MCB-1157876 to JR.

## Conflict of Interest Statement

The authors declare that the research was conducted in the absence of any commercial or financial relationships that could be construed as a potential conflict of interest.
